# Differentiation in Quinolone Resistance by Virulence Genotype in *Pseudomonas aeruginosa*

**DOI:** 10.1371/journal.pone.0042973

**Published:** 2012-08-08

**Authors:** Melissa Agnello, Annie Wong-Beringer

**Affiliations:** School of Pharmacy, University of Southern California, Los Angeles, California, United States of America; Centre National de la Recherche Scientifique, Aix-Marseille Université, France

## Abstract

*Pseudomonas aeruginosa* is a leading pathogen that has become increasingly resistant to the fluoroquinolone antibiotics due to widespread prescribing. Adverse outcomes have been shown for patients infected with fluoroquinolone-resistant strains. The type III secretion system (TTSS) is a major virulence determinant during acute infections through the injection of effector toxins into host cells. Most strains exhibit a unique TTSS virulence genotype defined by the presence of either *exoS* or *exoU* gene encoding two of the effector toxins, ExoS and ExoU, respectively. Specific TTSS effector genotype has been shown previously to differentially impact virulence in pneumonia. In this study, we examined the relationship between TTSS effector genotype and fluoroquinolone resistance mechanisms in a collection of 270 respiratory isolates. We found that a higher proportion of *exoU+* strains were fluoroquinolone-resistant compared to *exoS+* strains (63% vs 49%, p = 0.03) despite its lower overall prevalence (38% *exoU+* vs 56% *exoS+*). Results from sequencing the quinolone resistance determining regions (QRDRs) of the 4 target genes (*gyrA, gyrB, parC, parE*) indicated that strains containing the *exoU* gene were more likely to acquire ≥2 mutations than *exoS+* strains at MICs ≤8 µg/ml (13% vs none) and twice as likely to have mutations in both *gyrA* and *parC* than *exoS+* strains (48% vs 24% p = 0.0439). Our findings indicate that *P. aeruginosa* strains differentially develop resistance-conferring mutations that correlate with TTSS effector genotype and the more virulent *exoU+* subpopulation. Differences in mutational processes by virulence genotype that were observed suggest co-evolution of resistance and virulence traits favoring a more virulent genotype in the quinolone-rich clinical environment.

## Introduction

*Pseudomonas aeruginosa* is a gram-negative pathogen that causes opportunistic infections in susceptible hosts. It is a leading cause of acute pneumonia in hospitalized patients and is responsible for chronic lung infection in patients with cystic fibrosis. Its ability to cause both acute and chronic infections can be attributed to its broad arsenal of virulence factors. Specifically, the type III secretion system (TTSS) has been shown to be a major virulence determinant in the pathogenesis of acute infections.

*P. aeruginosa* utilizes the TTSS to deliver effector toxins (ExoS, ExoU, ExoY, and ExoT) directly into host cells, which can cause rapid cell necrosis or can modulate the actin cytoskeleton, allowing the pathogen to invade host cells and evade phagocytosis [1]. Depending on the disease site or background, genes encoding the cytotoxins ExoU and ExoS are present as variable traits and are mutually exclusive in most strains, the *exoS* genotype being the more prevalent of the two. The *exoS* genotype accounted for 72% of the 115 clinical and environmental isolates examined in one study while 28% of the strains contained the *exoU* gene. Specifically, a much lower proportion of *P. aeruginosa* obtained from the respiratory tract was comprised of *exoU+* strains than those from blood or wound (18% vs 40%, respectively) [2]. In a murine model of acute *P. aeruginosa* pneumonia, ExoU has been demonstrated to have the greatest impact on virulence relative to the other TTSS effector proteins (ExoS and ExoT) by measurement of mortality, bacterial persistence in the lung, and dissemination [4]. These experimental findings are supported by clinical studies where ExoU-secreting strains have been associated with poor outcomes of pneumonia [5] as well as persistence and severity of disease [6], [7]. More recently, El-Solh et al. [3] reported on the increased 30-day mortality of bacteremic patients infected with strains expressing the TTSS compared to those infected with non-TTSS expressing strains. Notably, none of the patients infected with an ExoU-secreting strain survived past 30 days.

The emergence of antibiotic-resistant *P. aeruginosa* strains has presented significant therapeutic challenges. The fluoroquinolone (FQ) antibiotics, ciprofloxacin and levofloxacin, exhibit potent *in vitro* anti-pseudomonal activity; however, due to its widespread use over the past decade as the most commonly prescribed antibiotic class to adults in the U.S., resistance has developed in parallel to its popularity in use [8], [9]. Of concern, a significant portion of the FQ-resistant strains are also multidrug-resistant [10], [11].

*P. aeruginosa* acquires resistance to the fluoroquinolones mainly through mutations in the quinolone resistance determining region (QRDR) of target genes essential for the topological maintenance of the genome, DNA gyrase and topoisomerase IV [12]–[14]. Both enzymes are composed of two subunits, encoded by the genes *gyrA, gyrB,* and *parC*, *parE*, respectively. DNA gyrase is responsible for catalyzing the negative supercoiling of DNA, and topoisomerase IV decatenates daughter replicons. Both functions are essential for successful replication of the genome. The reaction cycle includes breakage and resealing of double-stranded DNA. Fluoroquinolones bind to the enzymes and prevent the completion of the reaction, resulting in stalled replication forks and double stranded DNA breaks [15]. Mutations in the four target genes that confer resistance to the fluoroquinolones have been well described in *P. aeruginosa* [16], [17], with GyrA as the primary fluoroquinolone target [14], [18]. Other mechanisms of resistance include overexpression of efflux pumps as well as innate impermeability of the cell wall [19].

Previous work in our lab has shown that patients infected with FQ-resistant *P. aeruginosa* strains have 3-fold higher mortality and prolonged illness compared to those infected by susceptible strains [10]. In addition, we reported a significant association between FQ-resistance and the *exoU* TTSS genotype in 45 clinical isolates of *P. aeruginosa* obtained from various body sites exhibiting a range of susceptibilities to fluoroquinolones, suggesting a selection bias for development of FQ resistance based on TTSS effector genotype of the strains [20]. A recent study by Garey et al. analyzed bloodstream isolates of *P. aeruginosa* and found that *exoU+* strains were more frequently multi-drug resistant compared to *exoS+* strains [21]. Additional analysis on the data indicated that the highest frequency of resistance among the *exoU+* strains was towards the fluoroquinolones (87%, 13/15 vs 37%, 7/19 respectively, p = 0.0034). (personal communication) Similar results were observed by others examining corneal isolates [22]. Interestingly, Fleiszig et al has shown that resistance of *P. aeruginosa* to contact lens disinfectant was linked to acute cytotoxic activity from ExoU towards corneal epithelial cells and *exsA*, a transcriptional regulator of genes encoding effectors of the TTSS [23]. We hypothesized that *P. aeruginosa* strains, depending on their TTSS effector genotypes, differentially develop resistance-conferring mutations resulting in a selection bias for a FQ-resistant population predominated by *exoU*+ strains. Given the impact of ExoU on the outcomes of experimental and human lung infections, we tested our hypothesis in a large sample of clinical isolates obtained from respiratory sources; most of the patients from whom the isolates were obtained had pneumonia. We compared the following by TTSS effector genotype (*exoU* vs *exoS*): 1) prevalence and degree of FQ-resistance, 2) efflux pump overexpressed phenotype, and 3) mutations in target genes conferring FQ resistance.

## Materials and Methods

### Ethics Statement

This study has been approved by the institutional review boards at both Huntington Hospital and University of Southern California. Informed consent was waived from all participants since bacteria cultures were saved as part of a longitudinal epidemiological surveillance study for resistance trend. Respiratory cultures from hospitalized patients growing *Pseudomonas aeruginosa* were later identified retrospectively from microbiology records. All data was analyzed anonymously.

### Susceptibility Testing and Sequencing for Target Site Mutations

Bacterial isolates were stored in cryovials at −80C until ready for testing. Clonality of the clinical isolates was assessed by random amplified polymorphic DNA polymerase chain reaction (RAPD-PCR) using a primer sequence published previously [24] (Table 1). Susceptibility testing to levofloxacin was performed by broth microdilution in 2-fold dilution at concentrations ranging from 0.25 to 128 µg/ml according to guidelines recommended by CLSI [25]. FQ resistance is defined as levofloxacin MIC ≥4 µg/ml. Both the TTSS effector genotype (*exoU* and *exoS*) [20], [26] and the QRDR region of respective target genes, *gyrA, gyrB, parC* and *parE* was assayed by polymerase chain reaction (PCR) using previously published primers and conditions [17], [27]. The latter was sequenced to identify active and silent mutations compared to wild-type strains PAO1 (*exoS+/exoT+/exoY+*) and PA103 (*exoU+/exoT+/exoY+*). Primer sequences are listed in Table 1. Efflux pump overexpressed phenotype was determined by use of a commercially purchased efflux pump inhibitor (EPI, MC-0228) (Sigma) previously shown to broadly inhibit the multidrug Mex efflux pumps of *P. aeruginosa* in which the fluoroquinolones are substrates [10], [28]. An overexpressed phenotype is defined as ≥8-fold decrease in levofloxacin MIC when tested in the presence of EPI at 20 µg/ml previously established based on the change in MICs between wild type and efflux pump knockout mutants [28].

**Table 1:** Primers used for PCR and sequencing.

Name	Primer Sequence (5′–3′)	References
GyrA	Forward: ttatgccatgagcgagctgggcaacgact Reverse: aaccgttgaccagcaggttgggaatctt	27
GyrB	Forward: gcgcgagatgacccgccgca Reverse: ctggcggaagaagaaggtcaaca	17
ParC	Forward: cgagcaggcctatctgaactat Reverse: gaaggacttgggatcgtccgga	27
ParE	Forward: cggcgttcgtctcgggcgtggtgaagga Reverse: tcgagggcgtagtagatgtccttgccga	17
ExoS	Forward: atggcgtgttccgagtca Reverse: aggtgtcggttcgtgacgtct	26
ExoU	Forward: ggcacatatctccggttccttc Reverse: tcaactcagctgccaaccatgc	26
RAPD 272	agcgggccaa	24

### Statistical Analysis

All respiratory isolates were grouped by TTSS effector genotype and compared for their degree of resistance to levofloxacin as measured by MIC, efflux pump involvement (magnitude of decrease in levofloxacin MIC in the presence of an efflux pump inhibitor), and number and type of target site mutations. Chi-square or Fisher's exact tests were used where appropriate. A p value ≤0.05 denotes statistical significance.

## Results

### Bacterial Strains

A total of 270 *P. aeruginosa* isolates obtained from respiratory specimens from different patients were evaluated in this study. Majority of the strains (93%) caused pneumonia while the remainder caused bronchitis or colonized the airways. About 20% of the patients with pneumonia had ventilator-associated pneumonia. Clonality was determined for most strains (n = 223); those strains carrying both or neither genes encoding ExoU and/or ExoS were excluded. Strains with identical band patterns were considered to be related and assigned a unique random amplified polymorphic type. The 63 FQ-resistant *exoU* strains assessed fell into 34 clonal groups, and the 27 FQ-sensitive *exoU* strains assessed fell into 17 clonal groups. Among the *exoS* isolates, the 65 strains analyzed fell into 37 groups, and the 68 FQ-sensitive strains analyzed are in 26 groups. Overall, there are no more than 9 isolates in any given clonal group (Table 2). Clonal distribution was similar between FQ-resistant and FQ-sensitive strains with 51% (65/126) and 45% (43/95) of strains belonging to unique RAPD types, respectively.

**Table 2:** RAPD Results.

*exoU+,* FQ-resistant		*exoU+,* FQ-sensitive		*exoS+,* FQ-resistant		*exoS+,* FQ-sensitive	
RAPD Group	No. of Isolates	RAPD Group	No. of Isolates	RAPD Group	No. of Isolates	RAPD Group	No. of Isolates
1	5	1	1	1	5	1	8
2	3	2	2	2	5	2	9
3	4	3	1	3	2	3	6
4	3	4	2	4	3	4	5
5	3	5	1	5	2	5	5
6	1	6	1	6	2	6	4
7	3	7	2	7	2	7	4
8	1	8	1	8	2	8	3
9	2	9	3	9	2	9	3
10	2	10	1	10	2	10	2
11	1	11	2	11	1	11	2
12	3	12	1	12	1	12	2
13	1	13	2	13	1	13	2
14	1	14	1	14	1	14	1
15	4	15	2	15	1	15	1
16	1	16	2	16	1	16	1
17	1	17	3	17	1	17	1
18	4			18	1	18	1
19	4			19	1	19	1
20	2			20	1	20	1
21	1			21	1	21	1
22	1			22	1	22	1
23	1			23	1	23	1
24	1			24	1	24	1
25	1			25	1	25	1
26	1			26	1	26	1
27	1			27	1		
28	1			28	1		
29	1			29	1		
30	1			30	1		
31	1			31	1		
32	1			32	1		
33	1			33	1		
34	1			34	6		
				35	2		
				36	2		
				37	5		
SUMMARY: 63 strains in 34 groups; ranging 1 to 5 strains belonging to any one group.		SUMMARY: 27 strains in 17 groups; ranging 1 to 3 strains belonging to any one group.		SUMMARY: 65 strains in 37 groups; ranging 1 to 6 strains belonging to any one group.		SUMMARY: 68 strains in 26 groups; ranging 1 to 9 strains belonging to any one group.	

Note: Isolates were obtained from respiratory specimens from hospitalized patients spanning 2001–2009. RAPD Groups are designated a unique number under each TTSS-FQ categories and are not interchangeable (i.e. Strains under RAPD Group 1 ExoU-Resistant are different from strain(s) under the same RAPD Group number under ExoU-Sensitive, ExoS-Resistant, and ExoS-Resistant categories respectively).

### Prevalence and Degree of FQ resistance

**Figure 1:**
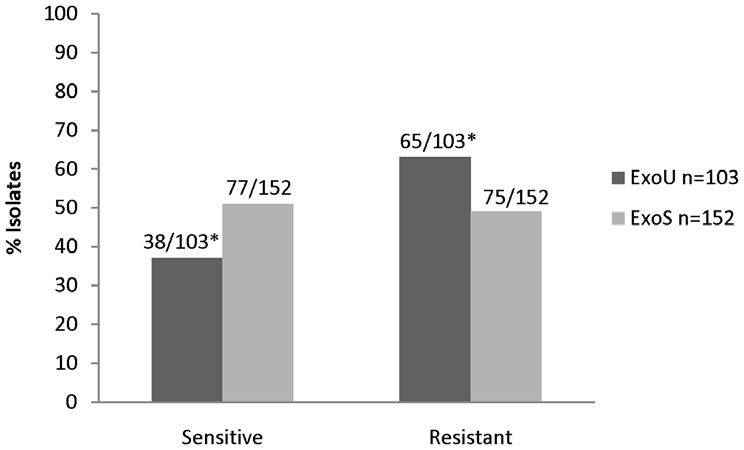
Frequency of fluoroquinolone-resistance among *exoU* vs. *exoS* containing isolates. Fluoroquinolone (FQ) resistance is defined as levofloxacin MIC ≥4 ug/ml. FQ-susceptible population is predominated by *exoS*+ isolates while FQ-resistant population is predominated by *exoU+* isolates. *p = 0.03.

Overall, less than half of the 270 isolates (38%, 103/270) were *exoU+* while 56% (152/270) were *exoS+*; five isolates were positive for both effector genes (2%) while 10 (6%) had neither. Similar proportion of *exoU+* and *exoS+* strains caused pneumonia (85%, 87% respectively). Slightly over half (54%, 145) of the isolates were FQ-resistant. The distribution of MIC values was similar between *exoU+* and *exoS+* populations. MIC50 and MIC90 of all 270 clinical strains were 8 and 64 µg/ml, respectively. Notably, a significantly higher proportion of *exoU+* strains were FQ-resistant compared to *exoS+* strains (63%, 65/103 vs 49%, 75/152; p = 0.03). (Figure 1).

### EPO Phenotype

Most FQ-resistant strains (92%, 133/145) exhibit efflux pump overexpressed (EPO) phenotype; only 8 strains had <8-fold decrease in MICs. A similar proportion of *exoU+* strains exhibit the EPO phenotype compared to *exoS+* strains (97%, 63/65 vs. 91%, 68/75 respectively; p = 0.13). The addition of an efflux pump inhibitor reduced the MIC in 59% (n = 145) of FQ-resistant strains to “susceptible” range at MIC <4 µg/ml overall; including 55% (n = 65) of the *exoU+* strains and 71% (n = 75) of *exoS+* FQ-resistant strains.

### Target Site Mutations

**Figure 2:**
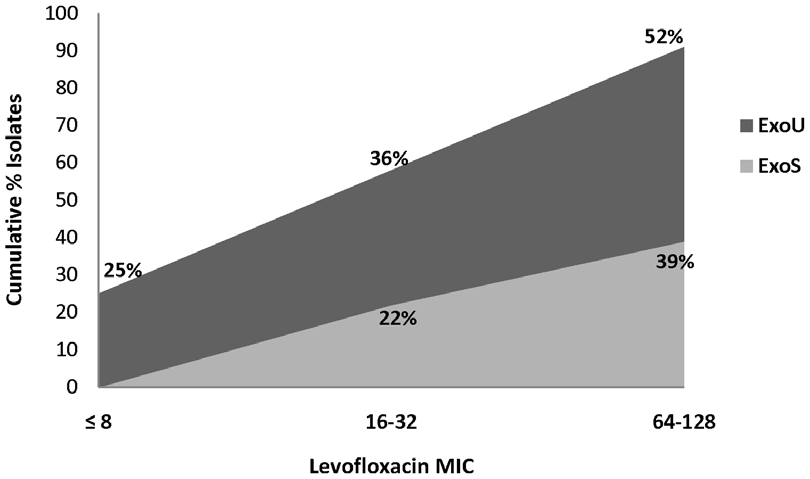
Cumulative Frequency of Isolates with ≥2 target site mutations at each given MIC comparing *exoU*- vs *exoS*-containing isolates. The proportion of isolates that has acquired at least two target site mutations is greater with *exoU+* than *exoS*+ isolates and increases in a linear fashion as MIC increases, with *exoU+* isolates starting at lower MICs. At a levofloxacin MIC ≤8 ug/ml, 25% of *exoU+* isolates compared to 0% of *exoS*+ isolates have 2 or more TSMs whereas for isolates with MICs ranging from 16–32 ug/ml, 36% *exoU+* isolates compared to 22% of *exoS+* isolates, and for isolates with MICs ranging from 64–128 ug/ml, 52% *exoU+* isolates compared to 39% of *exoS+* isolates have 2 or more TSMs.

The QRDR of the 4 target site genes (*gyrA, gyrB, parC,* and *parE*) was amplified by PCR and sequenced for resistance-conferring target site mutations (TSMs) in a subset of 108 isolates; 59 (55%) of which were *exoU+* and 49 (45%) were *exoS+* isolates. Table 3 summarizes all 108 isolates that have been sequenced for target site mutations. In general, MIC increased as the number of active mutations increased. Out of all the isolates with at least one target site mutation, only 4 had levofloxacin MIC <4 µg/ml. A total of 5 *exoU+* and 8 *exoS+* strains had no target site mutations; all showed the EPO phenotype. Among the 91 isolates with active mutations, a trend towards more single TSM was observed in *exoS+* strains (54%, 22/41 vs 38%, 19/50; p = 0.135) while more *exoU+* strains had combined TSMs (62%, 31/50 vs 46%, 19/41; p = 0.135). Interestingly, *exoU+* strains were more likely to acquire 2 or more TSMs than *exoS+* strains at lower MICs (≤8 µg/ml), 13% (4/31) vs. none. (Figure 2).

**Table 3:** Comparison of target site mutations (TSMs) by type III secretion effector genotype.

Target Protein	Amino acid substitution	*exoU* n = 59 (%)	*exoS* n = 49 (%)
*No TSM*		9/59 (15)	8/49 (16)
*Single TSM*		19/59 (32)	22/49 (45)
**GyrA**		**17/19 (89)**	**19/22 (86)**
	Thre83Ile	14	16
	Asp87Asn	3	2
	Lys65Arg	0	1
**GyrB**		**1/19 (5)**	**1/22 (4)**
	Glu468Asp	1	0
	Ser266Tyr	0	1
**ParC**		**1/19 (5)**	**1/22 (4)**
	Ser87Leu	1	1
**ParE**		**1/19 (5)**	**1/22 (4)**
	Asp419Asn	0	1
*Combined TSMs*		31/59 (53)	19/49 (39)
**GyrA**		**1/31 (3)**	**1/19 (5)**
	Thr83Ile+Asp87Glu	1	0
	Thr83Ile+Asp87Tyr	0	1
**GyrA+GyrB**		**1/31 (3)**	**2/19 (10)**
	Thr83Ile+Glu468Asp	1	2
**GyrA+ParC**		**27/31 (87)^a^**	**11/19 (58)^a^**
	Thr83Ile+Ser87Leu	19/27 (70)	3/11 (27)
	Thr83Ile+Glu91Lys	5/27 (19)	8/11(72)
	Thr83Ile, Asp87Asn+Ser87Leu	3/27(11)	0
**GyrA+ParE**		**1/31 (3)^b^**	**4/19 (21)^b^**
	Thr83Ile+Ile463Phe	1	4
**GyrA+ParC+ParE**		**1/31 (3)**	**1/19 (5)**
	Thr83Ile+Ser87Leu+Ile463Phe	1	1

NOTE: ^a^ p = 0.0189; ^b^ p = 0.041; chi-square test.

Substitution of isoleucine for threonine at position 83 in *gyrA* was the most commonly noted mutation among those with active mutations (88%, 80/91) and in similar proportion between *exoU+* and *exoS+* isolates (90%, 45/50 vs. 85%, 35/41, respectively). Of those with only this mutation at position 83 of *gyrA,* all except one had levofloxacin MIC ≥8 µg/ml. Even after addition of the EPI, about 37% (11/30) of those isolates remain resistant, with MIC ranging from 4 to 16 µg/ml. A total of 5 isolates have a different *gyrA* amino acid substitution, asparagine for aspartic acid at position 87. This mutation contributed only moderately to resistance in strains with combined TSM and EPO phenotype; levofloxacin MICs ranged from 1–16 µg/ml in those isolates, which lowered to ≤0.5 µg/ml after addition of EPI. In total, only 2 isolates each had any active mutations in *gyrB*, *parC*, or *parE* without a concurrent *gyrA* mutation; the corresponding levofloxacin MICs after addition of EPI for those isolates were: *gyrB* (2, 0.25 µg/ml), *parC* (8 µg/ml in both), and *parE* (2 µg/ml in both).

Notable differences in specific TSMs (single or combined) were observed between *exoU*+ and *exoS*+ isolates. Mutations unique to the *exoU+* strains included a double amino acid change in GyrA at positions 83 and 87, a single substitution at position 468 in GyrB, and a triple mutant with two GyrA changes (positions 83 and 87) and a ParC substitution at position 87. Mutations unique to the *exoS+* isolates result in a change from lysine to arginine at position 65 of GyrA, and a double *gyrA* mutant similar to the one found in the *exoU+* strains at positions 83 and 87. One *exoS+* isolate also carried a unique mutation in *gyrB* which resulted in a substitution of tyrosine for serine at position 466. Unlike any *exoU+* isolate in this study, one *exoS+* isolate had a single mutation in *parE* only, which resulted in a levofloxacin MIC of 16 µg/ml and 2 µg/ml in the presence of EPI.

Interestingly, among isolates with combined TSMs, mutation in *gyrA* is present in all isolates, but the proportion of *exoU+* and *exoS+* strains differ significantly in the subunit of topoisomerase IV affected. (Table 2) Strains containing the *exoU* gene were twice more likely to have combined mutations in both *gyrA* and *parC* than *exoS+* strains (48%, 28/59 vs. 24%, 12/49, p = 0.0439, OR 2.369 (95%CI: 1.014–5.536). On the other hand, *exoS*+ strains were more likely to have combined mutations in *gyrA* and *parE*.

Many silent mutations were also found in the target genes; *gyrB* and *parE* were the most affected, with 84% of all the isolates carrying one or more silent mutations in *gyrB* and 86% carrying one or more in *parE*. Silent mutations occur more frequently in the *gyrA* gene among *exoS+* than *exoU+* isolates (74%, 36/49 vs. 20%, 12/59; p<.0001). On the contrary, significantly more *exoU+* isolates have silent mutations in the *parE* gene than *exoS+* isolates (93%, 55/59 vs. 78%, 38/49. p = 0.019). The presence or number of silent mutations did not correlate with levofloxacin MIC.

## Discussion

Clinical isolates of *P. aeruginosa* carrying the gene encoding for either the ExoU or the ExoS TTSS effector proteins have been documented to differentially impact virulence in pneumonia as well as in the development of antimicrobial resistance. Compared to *exoS+* strains, *exoU+* strains have been shown to have higher cytotoxicity correlated with increased resistance, in both *in vitro* [20], [23] as well as *in vivo* murine models of infection [6], [29]. In a study of population structure *P. aeruginosa* isolates in Europe, the *exoU+* genotype was found to be significantly associated with multidrug resistance and ciprofloxacin resistance compared to the *exoS+* genotype [30]. The present study sought to explore the observed link between TTSS virulence and fluoroquinolone resistance in *P. aeruginosa*. We showed that *exoU+* and *exoS+* strains differentially acquire resistance-conferring mutations and that *exoU+* strains may be genetically favored in a fluoroquinolone-rich environment that stemmed from heavy prescribing.

*P. aeruginosa* develops resistance to the fluoroquinolones primarily from acquiring target site mutations or overexpression of multidrug efflux pumps from the Resistance-Nodulation-Division (RND) family [31]. Confirming our previously published findings, we have shown here that the fluoroquinolone-resistant subpopulation is predominated by *exoU+* strains in a large sample of respiratory isolates, despite the higher prevalence of *exoS+* strains overall. This may be related to our observation that *exoU+* strains more readily acquire multiple target site mutations and at a lower MIC than *exoS+* strains. Overexpression of multidrug efflux pumps belonging to the RND family of proteins results from mutations in genes that encode for the pumps or in genes that regulate their expression [31]. Here, we utilized an efflux pump inhibitor to assess the extent of efflux pump involvement in resistance by comparing the MICs of levofloxacin before and after the addition of efflux pump inhibitor. Efflux pump-overexpression significantly contributed to FQ-resistance in both *exoU* and *exoS*-containing isolates.

With respect to target site mutations, those observed in our isolates are consistent with previously published mutations [13], [14], [17], [27], [32]. Substitution of isoleucine for threonine at position 83 in GyrA is the most frequent TSM in our cohort of FQ-resistant isolates. Higher MICs are associated with an accumulation of mutations, the most common being a double mutation in *gyrA* and *parC*. Others have found strains with a double *gyrA-parC* mutation to be 3–4 times more resistant to ciprofloxacin than strains with a single mutation in *gyrA* [12]. Similar results were found in *Salmonella* sp. where mutations in *gyrA* and *parC* as well as double mutations in *gyrA* conferred high level resistance [33]. Our results provide further support for this stepwise accumulation of mutations mechanism [27]; only 2 resistant isolates have single mutations in *parC* without the “first step” threonine 83 mutation in *gyrA*. This shows that although GyrA is the main target of fluoroquinolones, a mutation in *gyrA* is not a strict prerequisite for the acquisition of mutations at other target genes leading to resistance. Despite previous results describing single mutations in the *gyrB* and *parE* genes not conferring much resistance [32], we found relatively high levofloxacin MICs ranging from 8–32 µg/ml (0.25–2 µg/ml in the presence of an EPI) in 2 isolates with a single *gyrB* mutation and one with a single *parE* mutation.

One striking difference observed between TTSS genotype in FQ resistance relates to the presence of specific active target site mutations conferring resistance. Isolates containing the *exoU* gene were twice as likely to have mutations in both *gyrA* and *parC* than *exoS+* isolates. Furthermore, the type of mutations in the *parC* gene differed with respect to TTSS effector genotype. The serine to leucine change at amino acid 87 in ParC was more likely to occur in *exoU+* strains, while the glutamate to lysine change at position 91 was more likely to occur in *exoS+* strains. This observed difference may point to a differential adaptive response of *exoU+* and *exoS+* to fluoroquinolone exposure.

Synonymous mutations have also been shown to have an effect on gene expression. When a library of green fluorescent protein genes that differed only in codon usage but not in the amino acid sequence was expressed, the levels of expression varied among the polymorphic genes, indicating that cellular processes can be affected without changing the amino acid sequence [34]. This is likely a result of a change in the mRNA secondary structure. Interestingly, we observed in this study differences in the prevalence and type of silent mutations that occurred in the QRDRs of the four target genes by TTSS effector genotype. The *gyrB* and *parE* genes had the highest number of silent mutations possibly because they are not the main contributors to enzymatic action and therefore can tolerate the polymorphisms. *exoS*-containing strains have a significantly higher rate of silent mutations in *gyrA* than *exoU+* strains while the opposite is observed for the *exoU+* strains with the *parE* gene; both TTSS effector genotypes have similar rates of silent mutations in *gyrB* and *parC*.

The results presented in this study show that resistance-conferring mutations developed differently in *exoU+* and *exoS+* strains. We speculate that the mechanism for this difference may involve the SOS response, a global response that allows bacteria to tolerate sudden increases in DNA damage. Fluoroquinolones induce the SOS response by poisoning the topoisomerase enzymes, leading to double stranded DNA breaks. Sub-MIC levels of ciprofloxacin have been shown to rapidly induce the SOS response in *S. typhimurium* [35]. A subset of genes induced by the SOS response include the error prone polymerases (Pol II, Pol IV, and PolV) [36]. These low-fidelity polymerases are able to perform translesion synthesis, enabling stalled replication forks to continue past DNA damage, but at the expense of introducing errors into the genome. By preventing induction of the SOS response *in vivo* and *in vitro*, pathogenic *E. coli* were unable to acquire resistance mutations [37]. This same study also showed that if any of the SOS-induced polymerases were made non-functional, the mutation rate was much less than in wild type. Relating those results to our findings leads us to question whether a difference in the SOS response in *exoU+* and *exoS+* strains could account for the differences in target site mutations observed. It is possible that differential quinolone exposure in patients from whom the strains were obtained may contribute to the difference in mutational processes between the two TTSS effector genotypes. We have minimized this potential bias by focusing only on respiratory isolates that caused acute infections in this study.

Differences in the target genes affected by resistance-conferring mutations in *exoU+* vs. *exoS+* strains may have important biological implications related to virulence expression. Promoters of certain genes have been shown to be especially sensitive to changes in the level of supercoiling, affecting expression levels. It has been proposed that supercoiling alters the structure of the promoter region, determining its accessibility to the transcription machinery [38]. Altered levels of supercoiling via inhibition of gyrase were shown to alter the expression levels of virulence genes in *Salmonella typhimurium* [39] as well as *Staphylococcus aureus* [40], suggesting that transcription of virulence genes may be regulated through changes in supercoiling. Others have shown that controlling the topology of the genome can be a mechanism of global gene regulation. An analysis of expression levels after exposure to a gyrase inhibitor in *Streptococcus pneumoniae* showed the genome to be organized in topology-reacting gene clusters [41]. The levels of supercoiling in fluoroquinolone-resistant *E. coli* strains with various target site mutations have been shown to differ [42] while altered protein abundances were observed among strains of *E. coli* with mutations in the gyrase gene compared to wild type [43]. Taken together, it is tempting to speculate that the different target site mutations in *exoU+* and *exoS+* strains could potentially affect topoisomerase activity and lead to differential expression of virulence genes with topology-sensitive promoters.

We present here evidence of differentiation in quinolone resistance by virulence genotype in a large sample of respiratory isolates of *P. aeruginosa*. A limitation of the present study is a lack of data regarding prior antibiotic exposure. The retrospective nature of the clinical component of the study is limited by the inconsistent documentation of antibiotic exposure in patients' medical charts. It is possible that prior antibiotic exposure in the affected patients drove the development of resistance in these strains during the course of infection; alternatively, acquisition of these resistant strains causing acute infections in these patients may be through horizontal transmission. Unpublished data from our lab testing *exoU+* (PA103 and one clinical isolate) and *exoS+* strains (PAO1 and one clinical isolate) serially passaged under increasing FQ concentrations indicates that the target site mutations accumulated in the resistant mutants *in vitro* were primarily GyrA mutation and do not mimic those that were observed in the large sample of clinical isolates that we surveyed in this study. The discordance in resistance development between *in vivo* and *in vitro* conditions likely reflects the complex *in vivo* environment that impacts the mutational process in ways beyond drug exposure. Future experiments using isogenic strains are planned to confirm whether the selection for resistant *exoU+* subpopulations imposed by heavy fluoroquinolone use is a form of genotype-by-environment interaction to ensure fitness of *Pseudomonas* evolving in a quinolone-rich environment. Importantly, negative outcomes reported in the literature in association with fluoroquinolone resistance in *Pseudomonas* infections may be attributable in part to the shift in population dynamics in which resistance and virulence traits co-evolved, favoring the more virulent *exoU+* strains. The current practice of widespread fluoroquinolone prescribing has far reaching negative consequences beyond antimicrobial resistance.
